# Exploring the interplay between emotional attitudes towards diabetes, eating behaviour and glycaemic control in patients with type 2 diabetes mellitus

**DOI:** 10.1017/S1368980024002179

**Published:** 2024-10-23

**Authors:** Olívia Garbin Koller, Tamires Freire de Carvalho Santana Andrade, Antônio Bonfada Collares Machado, Jessica Pinto Polet, Bárbara Pelicioli Riboldi, Cíntia Corte Real Rodrigues, Jussara Carnevale de Almeida

**Affiliations:** 1 Post-graduate Program in Nutrition, Food and Health, Universidade Federal do Rio Grande do Sul, Porto Alegre, Brazil; 2 Post-graduate Program in Medical Sciences: Endocrinology, Universidade Federal do Rio Grande do Sul, Porto Alegre, Brazil; 3 Post-graduate Program in Psychology, Universidade Federal do Rio Grande do Sul, Porto Alegre, Brazil; 4 Nutrition Division, Santa Casa de Misericórdia de Porto Alegre, Porto Alegre, Rio Grande do Sul, Brazil; 5 Nutrition and Dietetics Division, Hospital de Clínicas de Porto Alegre, Rua Ramiro Barcelos 2350, 1^o^ andar do bloco A, Porto Alegre PO 90035-003, Brazil; 6 Department of Nutrition, Faculdade de Medicina, Universidade Federal do Rio Grande do Sul, Porto Alegre, Brazil

**Keywords:** Diabetes mellitus, Type 2, Attitude, Eating behaviour, Nutrition education

## Abstract

**Objective:**

This study aimed to assess the association between emotional attitudes towards diabetes, eating behaviour styles and glycaemic control in outpatients with type 2 diabetes.

**Design:**

Observational study.

**Setting:**

Endocrinology Division of Hospital de Clínicas de Porto Alegre, Brazil.

**Participants:**

Ninety-one outpatients diagnosed with type 2 diabetes. Baseline assessments included data on clinical parameters, lifestyle factors, laboratory results, eating behaviour styles and emotional attitudes. All patients received nutritional counseling following diabetes recommendations. A follow-up visit was scheduled approximately 90 days later to evaluate changes in weight, medication dosages and glycated Hb (HbA1c) values. Patients were categorised based on their emotional attitude scores towards diabetes (positive or negative), and their characteristics were compared using appropriate statistical tests.

**Results:**

At baseline, no differences were observed in the proportion of patients with good glycaemic control, eating behaviour styles and emotional attitudes. However, patients with a positive attitude towards the disease exhibited a significantly better response in glycaemic control compared with the reference group (OR = 3·47; 95 % CI = 1·12, 10·75), after adjusting for diabetes duration, sex and medication effect score. However, when BMI was included in the model, the association did not reach statistical significance. Therefore, these results should be interpreted with caution.

**Conclusions:**

Patients with a positive attitude towards diabetes showed a greater reduction in HbA1c levels following nutritional counseling. However, baseline BMI could be a potential confounding factor.

Diabetes is a multifaceted chronic condition, necessitating ongoing medical attention and multifactorial risk reduction strategies beyond glycaemic control^(1)^. Its prevalence increases with age, and in 2021, Brazil ranked sixth globally, with 15·7 million people affected^(2)^. Continuous education and support for diabetes self-management are imperative to mitigate acute complications and lessen the risk of long-term complications. Substantial evidence supports various interventions aimed at enhancing diabetes outcomes. Structured behavioural therapy, encompassing a low-energy meal plan and regular physical activity, holds paramount importance for individuals at heightened risk of developing overweight or obese among patients with type 2 diabetes (T2DM)^(1)^. Consistent with national standards for diabetes education and support, individuals with diabetes should engage in diabetes self-management education and receive requisite support to enhance knowledge, decision-making abilities and proficiency in essential diabetes self-care skills^(3)^.

Attitude can be regarded as a marker of awareness and reflects the individual’s decision to adopt or reject and sustain certain behavioural standards. This aspect warrants further investigation across diverse populations and cultural groups^(4–6)^. Increased population awareness plays a crucial role as a determinant in the prevention of diabetes and its associated complications, along with related metabolic disorders such as overweight and hyperglycaemia^(7)^. The commitment to adhere to or the inclination to discontinue treatment, which reflects a positive or negative attitude towards the disease, is a constant aspect in the daily lives of patients with diabetes^(8)^.

The Diabetes Attitudes Questionnaire (ATT-19) is a self-administered questionnaire designed to evaluate the psychological and emotional dimensions related to diabetes^(7)^. The cross-cultural adaptation of the ATT-19 was conducted using a sample of Brazilian T2DM patients, demonstrating the suitability of the instrument to the Portuguese language and the cultural context of Brazil^(8)^. In a cross-sectional study involving 202 patients with T2DM from Recife, Brazil, it was noted that 85·6 % of them exhibited a negative attitude towards the disease. Additionally, all forty-two patients (100 %) with poor glycaemic control demonstrated a negative emotional attitude, compared with 81·3 % of patients with good glycaemic control^(9)^. Moreover, an increase in positive attitudes towards diabetes may lead to improved glycaemic control and body weight among patients with T2DM, as evidenced by findings from a randomised clinical trial^(10)^.

Stress or negative attitudes can influence the self-control of patients who need to restrict their food intake, such as individuals with diabetes^(11)^ or overweight^(12)^. Individuals may turn to eating instead of addressing unpleasant emotions due to the cognitive burden that overwhelms self-regulation resources^(13)^. Among patients with T2DM, research has indicated associations between elevated levels of diabetes-related stress and unhealthy eating behaviours, such as the consumption of fast foods and soft drinks^(14)^, as well as restrained, external and emotional eating behaviours^(15)^. Nevertheless, studies examining the associations among emotional attitudes towards diabetes, eating behaviour styles and glycaemic control in patients with T2DM remain limited.

In this regard, the present study posits that patients with T2DM who exhibit a positive attitude towards the disease may demonstrate healthier eating patterns and better control of parameters related to diabetes compared with those with a negative attitude. Therefore, the study aims to assess the potential association between emotional attitudes towards diabetes, eating behaviour styles and glycaemic control in patients diagnosed with T2DM.

## Material and methods

### Study population

The present observational study was conducted among patients diagnosed with T2DM, defined as individuals aged over 30 years at the onset of diabetes, with no prior episodes of ketoacidosis or documented ketonuria and who had not initiated insulin therapy within the 5 years following diabetes diagnosis^(16)^.

The study recruited outpatients who consecutively attended the Endocrinology Division of the Hospital de Clínicas de Porto Alegre, Brazil. Exclusion criteria included a diagnosis of maturity-onset diabetes of the young, having received dietary counseling from a nutritionist for at least 6 months prior to the study, age over 80 years, pregnancy and/or lactation, urinary tract infection or other renal disease (except diabetic kidney disease), severe liver disease, decompensated heart failure or any acute or terminal illness, cancer and/or wasting syndrome. Patients with cognitive, neurological or psychiatric conditions that impaired their ability to answer questions (as determined by the researcher) were also excluded.

### Clinical evaluation

Patients underwent clinical, laboratory and lifestyle evaluations between September 2017 and February 2020. Clinical data, including duration of diabetes, comorbidities associated with the disease and medication use, were extracted from the patients’ electronic medical records during the medical visit closest to the nutritional assessment date.

Patients were categorised as current or non-current smokers (former and non-smokers) and self-identified as white or non-white. Socio-economic status was assessed using a standardised Brazilian questionnaire^(17)^, which categorised individuals into strata A to E (with A representing the highest status). The classification considers the possession of certain material goods, the education level of the household head, the presence of domestic employees and the availability of public services at the residence. Patients in strata C1, C2 or D/E were classified as low income. The short version of the International Physical Activity Questionnaire was utilised to evaluate activities performed over a typical week in minutes, converted to metabolic equivalent tasks^(18)^. Patients reporting activities equivalent to at least 600 metabolic equivalent tasks per week were categorised as having a moderate physical activity level, while those reporting lower metabolic equivalent tasks were classified as sedentary^(19)^.

Patients with an estimated glomerular filtration rate < 60 ml/min/1·73 m^2^ and/or persistently elevated urinary albumin excretion, defined as albuminuria ≥ 14 mg/dl, were categorised as having diabetic kidney disease^(20)^. Blood pressure was measured twice, following a 5-minute rest, using a digital sphygmomanometer (Omron HEM-705CP) in accordance with international recommendations^(21)^. Hypertension was defined as systolic blood pressure > 140 mmHg or diastolic blood pressure > 90 mmHg measured on two occasions and/or the use of antihypertensive drugs^(22)^.

A medication score was utilised to evaluate the overall use of antidiabetic agents (both oral and insulin). Initially, the percentage of the maximum daily dose for each medication was calculated. Subsequently, this percentage was multiplied by an adjustment factor corresponding to the median absolute decrease in glycated Hb (HbA1c) associated with each agent. These products were then aggregated to compute the final Medication Effect Score. A score of ≥ 100 was considered when the maximum dose of medication was reached^(23)^. The oral antidiabetic medications included in this patient sample were metformin, glyburide, gliclazide, saxagliptin, sitagliptin, dapagliflozin and empagliflozin.

Blood samples were collected following a 12-hour fast, and all analyses were conducted at the Laboratory Diagnostic Service of the Hospital de Clínicas de Porto Alegre, Brazil. HbA1c was measured by HPLC in a Variant II Turbo System (reference range 4·0–6·0 %); plasma glucose was quantified by the enzymatic colorimetric method (Roche Diagnostic); serum values of TAG, total cholesterol and HDL-cholesterol levels were measured by enzymatic colorimetric methods. LDL-cholesterol was calculated using the Friedewald equation (LDL-cholesterol = total cholesterol – HDL-cholesterol – TAG/5)^(24)^ only in patients with TAG values < 400 mg/dl. Serum creatinine was measured by the Jaffé method. Glomerular filtration rate was estimated by the Chronic Kidney Disease Epidemiology Collaboration Calculator, and urinary albumin excretion was measured by immunoturbidimetry. Patients were considered within the therapeutic target according to the following criteria: fasting plasma glucose values < 130 mg/dl; HbA1c values < 7 %; serum TAG < 150 mg/dl; serum HDL-cholesterol > 40 mg/dl for men and > 50 mg/dl for women; LDL-cholesterol < 100 mg/dl^(25)^.

### Nutritional assessment

Patients underwent anthropometric measurements, dietary assessment, evaluation of eating behaviour and assessment of emotional attitude. All questionnaires used in this study were administered through interviews, where the evaluator verbally posed questions and the respondent provided answers based on their knowledge. Anthropometric measurements included weight, height, and waist circumference. Weight and height were measured with patients barefoot and wearing light clothing, using a Welmy® mechanical scale with a capacity of 150 kg. Waist circumference was measured once to the nearest 1 cm, at the midpoint between the lower costal margin and the iliac crest, near the navel, using a flexible, non-elastic fibre glass tape. BMI was calculated using the formula weight (kg)/height (m)^2^, with a target value of < 25 kg/m^2^ for adults^(26)^ and < 27 kg/m^2^ for older adults^(27)^. Higher waist circumference was defined as ≥ 86 cm for women and ≥ 92 cm for men, according to the cut-off points of Brazilian population^(28)^.

*Food consumption* data were collected using a quantitative FFQ administered by a trained nutritionist. This FFQ was previously developed and validated with patients in Southern Brazil^(29,30)^. It comprises ninety-eight food items and captures dietary intake over the preceding 12 months. Additionally, patients were provided with a portfolio containing photographs of each food item and portion size to aid in identifying their consumed portions. The FFQ-derived intake data were converted into daily consumption to estimate nutritional composition using information from the Brazilian Food Consumption Table^(31)^.

### Eating behaviour assessment

The Portuguese version of the Dutch Eating Behaviour Questionnaire (DEBQ) was used to assess eating behaviour^(32)^. This questionnaire comprises thirty-three items and measures three eating behaviour styles: Restrained Eating (ten items), Emotional Eating (thirteen items) and External Eating (ten items). Participants respond to items using a five-point Likert scale ranging from ‘never’ (one point) to ‘very often’ (five points). A mean score was calculated for each subscale, with higher scores indicating higher levels of the respective behaviour.

### Emotional attitude towards diabetes assessment

The emotional attitude towards diabetes was evaluated using the Diabetes Attitudes Questionnaire (ATT-19)^(7)^, Portuguese version^(8)^, which demonstrated a Cronbach’s alpha of 0·91 and Kappa coefficients ranging between 0·44 and 0·69^(8)^. This questionnaire comprises nineteen items organised into six factors: (a) stress associated with diabetes; (b) receptivity to treatment; (c) confidence in treatment; (d) personal effectiveness; (e) health perception and (f) social acceptance. Responses are recorded on a five-point Likert scale ranging from ‘strongly disagree’ (one point) to ‘strongly agree’ (five points), with questions 11, 15 and 18 scored in reverse. The total score ranges from nineteen to ninety-five points, with a score > 70 indicating a positive attitude towards the disease^(8)^. While validation criteria have been consistently reported, the factor structure of the ATT-19 has not been stable and well -defined, suggesting the need for further analysis. Therefore, exploring the construct validity of the ATT-19 questionnaire in our patient sample was deemed important (see online Supplementary Material).

### Nutritional counseling provided and patient follow-up

Nutritional counseling was offered both at the baseline and the follow-up visit. Healthy body weight was determined based on the criteria of BMI < 25 kg/m^2^ for adults and < 27 kg/m^2^ for older participants^(26,27)^. The estimated energy requirement was calculated using the Institute of Medicine Equation with the actual weight^(33)^, and an energetic restriction of 500–1000 kcal was advised for patients who were overweight^(34)^. All patients received nutritional counseling in accordance with diabetes recommendations^(1)^, tailored to their usual consumption patterns as assessed by FFQ, employing a patient-centred approach^(1)^. Consumption of fruits, vegetables, low-fat dairy and whole grains was encouraged. Additionally, patients without medical contraindications were encouraged to engage in 150 min of physical activity per week^(34)^. A follow-up visit was scheduled within a 90-d period as per the service routine to verify weight, medication doses and HbA1c values.

### Statistical analysis

The normality of the variables was assessed using the Shapiro–Wilk test. Descriptive analysis of the variables was conducted, with continuous variables presented as means (standard deviations) or medians (interquartile ranges) depending on their distribution. Categorical variables were expressed as absolute and relative frequencies.

Patients were categorised based on their positive or negative emotional attitude towards diabetes, and their characteristics were compared using the *t* Student test, the Mann–Whitney *U* test, the *χ* ^2^ test or Fisher’s exact tests, as appropriate. Follow-up data were extracted from patients’ medical records, including weight and glycaemic control. The difference between the second measurement of HbA1c and the baseline was considered the variation in HbA1c. Patients were then categorised based on this change into those who experienced a reduction in HbA1c and those who had changes or increased values over time. The same procedure was applied to weight values.

Logistic regression models were employed to examine the associations between positive emotional attitude and the reduction of HbA1c, while adjusting for potential confounding variables selected based on univariate analyses or clinical relevance. In the initial analysis, the association between positive emotional attitude and the reduction of HbA1c (as the dependent variable) was assessed. The second analysis (model 1) was adjusted for diabetes duration and sex. The third analysis (model 2) included additional adjustment for the medication effect score. The fourth analysis (model 3) extended to model 2 by incorporating physical activity and BMI.

The statistical analyses were conducted using the SPSS 18.0 Statistical Package (PASW Inc.), with a process involving double entry and subsequent database cleaning. A significance level of *P* < 0·05 (two-tailed) was considered statistically significant for all analyses.

## Results

**Figure 1.**
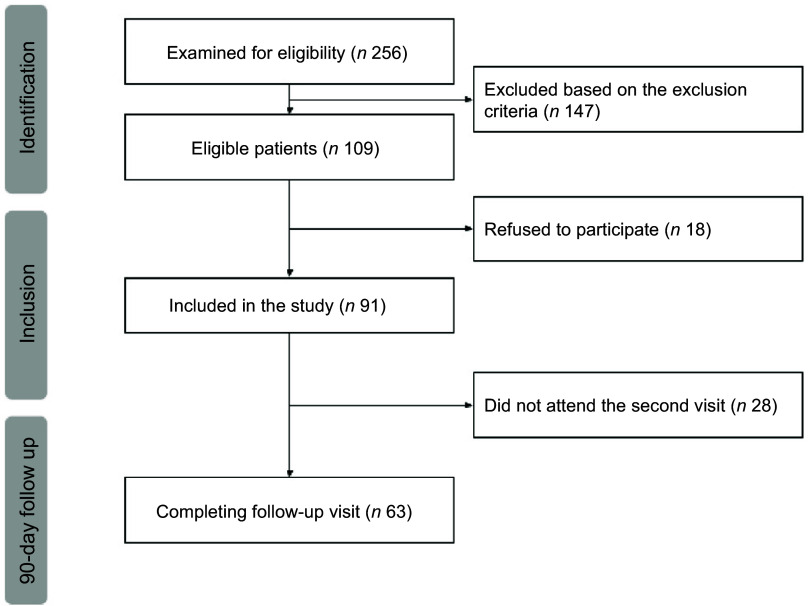
STROBE flow chart of participants included in the present analysis. STROBE, Strengthening the Reporting of Observational Studies in Epidemiology.

A total of 256 patients were screened, and 109 were deemed eligible over a 2-year period. Among them, ninety-one patients provided consent to participate in the study (flow chart in Fig. 1). The majority of the sample comprised women (*n* 51; 57 %) with a mean age of 60 (sd 9) years, a BMI of 32·7 (sd 6·6) kg/m^2^, a diabetes duration of 15 (sd 9) years and HbA1c values of 9·2 (sd 1·9) %.

Patients were categorised based on their emotional attitude: twenty-three patients (25 %) were classified as having a positive attitude, while sixty-eight patients (75 %) exhibited a negative attitude towards diabetes. Clinical characteristics, lifestyle and laboratory data of patients according to the ATT-19 are summarised in Table 1.

**Table 1.** Socio-demographic, clinical and nutritional characteristics of patients with type 2 diabetes considering negative or positive emotional attitude towards diabetes by ATT-19^(8)^ (*n* 91)

	Emotional attitude towards diabetes				
	Patients with positive attitude		Patients with negative attitude		*P*
	*n*	%	*n*	%	
*n*	23		68		
Age, years					
Mean	58		61		0·32^1^
sd	7		9		
Females	11	47·8 %	41	60·3 %	0·29^2^
Whites	17	73·9 %	51	75 %	0·91^2^
Socio-economic status: low income	17	73·9 %	41	60·3 %	0·24^2^
Years of study					
Mean	9		7		0·61^1^
sd	4		4		
Diabetes duration, years					
Mean	11		16		0·20^1^
sd	8		9		
Insulin and oral antidiabetics	21	91·3 %	57	83·8 %	0·37^2^
Maximum dose of antidiabetics agents, yes	20	87·0 %	60	88·2 %	0·87^2^
Hypertension	16	72·7 %	55	80·9 %	0·41^2^
Diabetes kidney disease	14	60·9 %	37	54·4 %	0·59^2^
Current smoking	3	13 %	26	38·2 %	0·07^3^
Physical activity: low level	17	73·9 %	60	88·2 %	0·18^3^
Nutritional counseling	12	52·2 %	25	67·6 %	0·19^2^
	Mean	sd	Mean	sd	
Anthropometric assessment					
BMI, kg/m²	31·7	6·7	33·1	6·6	0·63^1^
Higher waist circumference					
* n*	20		64		0·17^3^
%	87 %		95·5 %		
Estimated energy requirement, kcal/day	2162·3	354·3	2098·1	369·8	0·67^1^
Food consumption (FFQ)					
Total energy intake (TEI), kcal/day	2206·3	720·5	2107·1	1019·2	0·35^1^
Carbohydrates, %TEI	52·1	9·1	54·5	6·3	0·24^1^
Total lipids, %TEI	27·8	6·1	24·7	5·4	0·44^1^
Proteins, %TEI	20·1	4·3	20·9	3·7	0·48^1^
Laboratory values					
Fasting plasma glucose, mg/dl	161	58	176	70	0·35^1^
Glycated Hb, HbA1c, %	9·4	2·3	9·1	1·7	0·15^1^
Total cholesterol, mg/dl	170	42	171	46	0·71^1^
HDL-cholesterol, mg/dl	42	13	42	12	0·89^1^
LDL-cholesterol, mg/dl	81	39	86	39	0·85^1^
TAG, mg/dl					
Median	215		177		0·14^4^
IQR	160–261		120–245		
Dutch Eating Behavior Questionnaire, DEBQ					
Restrained eating, subscale	2·54	0·90	2·60	0·79	0·76^1^
Emotional eating, subscale	1·84	0·99	2·19	0·93	0·12^1^
External eating, subscale	2·41	0·69	2·76	0·76	0·05^1^

%TEI, percentage of total energy intake.

Data presented as mean (sd), median and interquartile range or number of patients for total cases (%).

^1^Test *t* of Student; ^2^ *χ* ^2^ test; ^3^Fisher’s exact test, ^4^U of Mann–Whitney test; maximum dose of antidiabetic agents was considered when the medication effect score was ≥ 100^(23)^·Low level of physical activity was considered when patients performed < 599 metabolic equivalent tasks per week of physical activities^(19)^; Higher waist was considered when waist ≥ 86 cm for women and ≥ 92 cm for men^(28)^.

A higher proportion of patients with a negative emotional attitude were classified as sedentary compared with patients with a positive emotional attitude (88 % *v*. 74 %). Additionally, the negative emotional attitude group had more current smokers (38 % *v*. 13 %) and patients with a higher waist circumference (91 % *v*. 74 %) compared with the positive emotional attitude group, although it did not reach statistical significance (*P* > 0·05). We did not observe differences in the proportion of patients with good glycaemic control between the two groups (*P* = 0·59). However, we observed a difference of 0·3 % in baseline HbA1c between patients with positive and negative attitudes towards diabetes, although it did not reach statistical significance (*P* = 0·15). Furthermore, no differences were observed in diabetes medication use or medication effect score between the two groups. In terms of DEBQ subscales, patients with a negative emotional attitude exhibited higher scores on the external eating dimension (*P* = 0·05) compared with the positive attitude group. However, no significant differences were observed in the total score of restricted eating (*P* = 0·76) or emotional behaviours (*P* = 0·12) between the negative and positive attitude groups. Other differences in clinical, basal laboratory and anthropometric values, total energy and macronutrient intake were also not observed.

**Figure 2.**
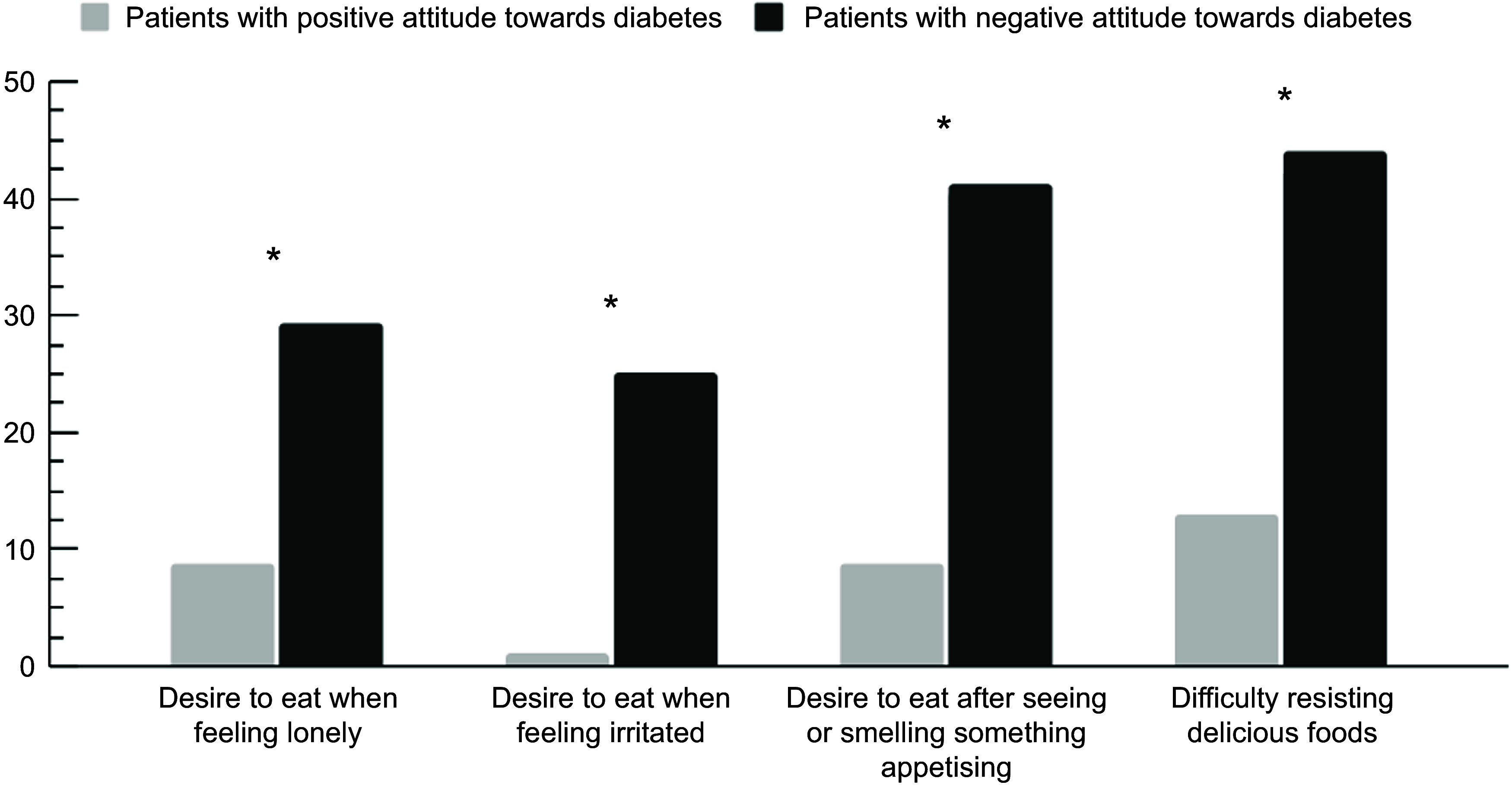
Proportion of patients with type 2 diabetes who reported emotional and external eating attitudes (often/very often) according to positive/negative attitude towards diabetes (*n* 91)· *P < 0·05 (*χ* ^2^ test).

The responses of patients with both positive and negative attitudes towards diabetes who reported emotional and external eating behaviours as ‘often’ or ‘very often’ were assessed by DEBQ items individually. These results are presented in Figure 2 and Table S2 in the online Supplementary Material. A higher proportion of patients with negative attitudes reported feeling like eating when they are feeling alone or irritated, if they see or smell something delicious, and having difficulty resisting eating delicious food compared with patients with a positive attitude towards diabetes (*P* < 0·05 for all comparisons).

After the baseline assessment, the follow-up visit for nutritional counseling was scheduled in a median of 114 (IQR 98–133) days. Among the ninety-one patients, sixty-three attended this second consultation with a nutritionist (69 %). However, information about a second measure of body weight and HbA1c values was obtained from the medical records of twenty-five out of twenty-eight missing patients (89 %) for follow-up analyses. In an exploratory analysis, twenty-one patients with a negative attitude (32 %) did not return for the follow-up visit, while only four patients with a positive attitude (17 %) did not return (*P* = 0·17). Regarding changes in HbA1c over a median of 143 (IQR 119–209) days, a smaller proportion of patients with a negative attitude (44 %) had a reduction in HbA1c values compared with the positive attitude group (71 %; *P* = 0·03). No differences in weight change (*P* = 0·10) or medication score for diabetes (*P* = 0·89) were observed between the emotional attitude groups during this period.

Table 2 presents the logistic regression model assessing the association between a positive emotional attitude towards diabetes and the reduction of HbA1c as the dependent variable. Patients with a positive emotional attitude had higher OR (3·47; 95 % CI: 1·12, 10·75) for HbA1c reduction over 4–7 months compared with those with a negative emotional attitude, after adjusting for diabetes duration, sex and medication effect score. However, when BMI and physical activity level were added to the model 3 adjustments, the association between a positive attitude towards diabetes and HbA1c did not reach statistical significance: OR = 2·86 (95 % CI: 0·89, 9·19).

**Table 2.** Logistic regression models were performed to evaluate the association between reduction of glycated Hb (as the dependent variable) and emotional attitudes in patients with type 2 diabetes (*n* 82)

Emotional attitude towards diabetes	Crude model		Model 1		Model 2		Model 3	
	OR	95 % CI	OR	95 % CI	OR	95 % CI	OR	95 % CI
Positive attitude	**3·14**	**1·08, 9·21**	**3·37**	**1·09, 10·38**	**3·47**	**1·12, 10·75**	2·86	0·89, 9·19

Bold values denote significance (*P* < 0·05).

Model 1 = adjusted for diabetes duration and sex.

Model 2 = adjusted for model 1 + medication effect score^(23)^.

Model 3 = adjusted for model 2 + low physical activity level^(19)^ and BMI.

## Discussion

The observational study investigated the potential link between emotional attitude towards diabetes, eating behaviour styles and glycaemic control in T2DM patients. Our findings indicate no discernible differences in eating behaviour styles between patients with positive and negative attitudes towards diabetes within our sample. However, upon analysing patient responses post-nutritional counseling, we observed that those harbouring a positive attitude had approximately three times higher likelihood of reducing HbA1c compared with counterparts with a negative attitude, even after accounting for some confounding variables.

In our sample of T2DM patients, a negative attitude towards diabetes was prevalent among the majority (78 %) of those interviewed. This finding aligns with results from similar studies conducted in primary health care settings in southeastern Brazil^(4,35)^. Diabetes, being a chronic condition, significantly impacts various aspects of individuals’ lives, diminishing their quality of life and potentially leading to reduced motivation for self-care management^(1)^. Furthermore, our study was conducted in a specialised setting, involving patients with complex diabetes management needs. Interestingly, patients with a negative attitude were more prone to missing appointments with nutritionists in our sample.

In our study, no significant differences were noted in eating behaviour styles (restrained, external and emotional eating) between patients with positive and negative attitudes towards diabetes. However, there was a higher proportion of patients with a negative attitude towards diabetes, who exhibited emotional and external eating behaviours, as per the DEBQ items. To our knowledge, this is the first study to explore the association between emotional attitude and eating behaviour styles measured by the DEBQ, which limits direct comparisons with existing literature. Nonetheless, it is crucial to acknowledge that diabetes distress can profoundly impact self-care practices, including dietary habits, and ultimately affect disease prognosis^(14,36,37)^. Diabetes distress encompasses a range of negative emotions such as sadness, fear, guilt and frustration, which must be addressed to achieve acceptance of the condition. When individuals experience these emotions, the expected reward associated with food consumption overrides cognitive control, leading to an increased drive to eat despite attempts to restrain intake^(38)^. Patients with a negative attitude towards diabetes reported higher instances of eating to alleviate negative feelings, being tempted by appetising sights or smells and struggling to resist indulging in delicious foods compared with those with a positive attitude towards diabetes. Consistent with findings from a cross-sectional study involving 183 patients with T2DM, diabetes-related and general stress can prompt individuals to exhibit restrained, external and emotional eating behaviours^(15)^.

Regarding glycaemic control, in contrast to findings reported by other authors^(9)^, we did not observe significant differences in the proportion of patients with good glycaemic control between the negative and positive emotional attitude groups, possibly due to our sample size limitations. However, we did note a slight difference of 0·3 % in baseline HbA1c levels between patients with positive and negative attitudes towards diabetes. Likewise, in exploratory analyses concerning changes in HbA1c levels, we identified an association between HbA1c reduction and a positive attitude towards diabetes following the initiation of nutritional counseling. While we did not find a directly comparable prospective study, our findings align with previous research^(10,39)^. Negative attitudes towards diabetes, characterised by perceptions of the condition as a social burden, are associated with greater challenges in implementing lifestyle modifications and adhering to self-management practices^(39)^. A prospective study involving Swedish patients with T2DM revealed that individuals with a more favourable attitude towards diabetes experienced significantly improved and sustained metabolic control following a year-long health education program, while those with negative attitudes showed no reduction in HbA1c levels^(40)^. Similarly, in a clinical trial involving Italian older adults with T2DM, participants randomised to an intensive individualised education programme demonstrated improved attitudes towards the disease, alongside reductions in HbA1c levels and BMI^(10)^. Adequate knowledge about diabetes is known to positively correlate with treatment adherence^(41)^, fostering a positive attitude towards the condition and facilitating a favourable metabolic response following self-care education. In our study, patients initiated the nutritional education process, but this hypothesis warrants further exploration through randomised clinical trials with larger sample sizes.

This study has several limitations inherent to its design and sample characteristics. First, the observational nature of the study precludes establishing causation, and residual confounding factors may still influence the results. Second, our sample is derived from a non-probabilistic selection of outpatients from a single hospital in Southern Brazil, limiting the generalizability of findings to broader populations. Third, the relatively modest sample size may lack the power to detect certain relationships, such as associations between emotional attitudes and eating behaviour styles, and thus these results should be interpreted with caution. Moreover, the scarcity of studies with similar objectives in the literature hinders robust comparisons with other contexts. Future investigations employing larger, more diverse samples are essential to confirm and extend our findings. Despite these limitations, we employed validated questionnaires to assess emotional attitudes towards diabetes, demonstrating adequate construct validity within our T2DM sample. Additionally, the Brazilian Portuguese version of the DEBQ exhibited satisfactory psychometric properties in a sample of Brazilian Portuguese-speaking adults, enhancing the reliability of our measures.

These findings underscore the significance of integrating psychological screening and support into diabetes care practices. Psychological and social challenges can significantly impede an individual’s ability to effectively manage diabetes^(1)^. Therefore, understanding patients’ attitudes towards their condition can inform targeted educational interventions during nutritional counseling, aimed at enhancing treatment adherence, quality of life and health outcomes. However, there remains a gap in understanding potential mediating variables between emotional attitudes and metabolic control in T2DM. Consequently, further research with robust designs is warranted to explore these associations comprehensively. Such studies could lay the groundwork for developing interventions tailored to fostering positive attitudes among patients with T2DM.

In summary, our findings suggest that a positive attitude was associated with a more significant reduction in HbA1c following nutritional counseling, compared with patients harboring a negative attitude towards their diabetes among our sample of T2DM outpatients. These results underscore the importance of evaluating emotional attitudes towards diabetes and associated factors in patients with T2DM.

## Supporting information

Koller et al. supplementary material Koller et al. supplementary material

## Supplementary material

For supplementary material accompanying this paper visit https://doi.org/10.1017/S1368980024002179
