# Supplementary material for: Exploring the interplay between emotional attitudes towards diabetes, eating behaviour and glycaemic control in patients with type 2 diabetes mellitus
Source: Public Health Nutr. 2024 Oct 23;27(1):e237. doi: 10.1017/S1368980024002179 (PMC11645113; doi:10.1017/S1368980024002179)
Supplement: Koller et al. supplementary material [file S1368980024002179sup001.docx]

**Supplementary Material**

**Exploring the Interplay between Emotional Attitudes toward Diabetes, Eating Behavior, and Glycemic Control in Patients with Type 2 Diabetes Mellitus**

**OBJECTIVE**

To explore the construct validity of the ATT-19 questionnaire in our patients sample.

**METHODS**

To explore the construct validity of ATT-19 questionnaire in our patients sample, we performed a Factorial Analysis. First, the factorability of the data was verified using the Kaiser-Meyer-Olkin (KMO) adequacy test^(1)^ and the Bartlett sphericity test^(2)^. Afterward, parallel analysis of principal components with polychoric correlations and parallel analysis using the minimum rank factorial analysis were used to determine the number of factors^(3,4)^. Polychoric correlations were used according to Likert Scale, in which the variables are ordinal^(5)^. Finally, extraction of factor loads and explained common variance was done by Minimum Rank Factor Analysis^(6)^. The oblimin rotation method was used in the extraction of loads, since it is expected that the scale factors present correlation^(7)^. These analyses were conducted in Software R version 4.0.5 (Team, R. C, 2013), using the psych package^(8)^.

**RESULTS**

Considering the construct validity of the ATT-19 scale, the data showed good factorability with a KMO = 0.74 and Bartlett's sphericity test significant (χ2(171) = 472.2, *p*<0.001). The parallel analysis scree plot (**Figure 1 in supplementary material**) indicated two factors for the scale, since the eigenvalue of the third factor was below the simulated eigenvalue of the parallel analysis^(9)^. It suggested the extraction of factor loadings for two factors, unlike the original study, which suggested a single factor^(10)^. The extraction of factorial loads was done by the Minimum Rank Factor Analysis, demonstrating items with factorial loads between 0.35 and 0.81 (**Table 1 in supplementary material**). In the present study, only factor loads above 0.32 and items that did not have cross loadings greater than 0.20 were retained^(2)^. Therefore, seven items were excluded (Q2, Q5, Q11, Q12, Q13, Q14, and Q15) from the scale. After removing each item^(11)^, the exploratory factorial analysis was carried out until a satisfactory solution for both factors was found. The first features alpha 0.79, and the second 0.70.

The regression diagnoses did not indicate any problem involving the collinearity of the measures, and the variance inflation factor (VIF) presented a value < 2 for all the variables in the model. However, the value of Cook's distance indicated the presence of influential outliers **(Figure 2 in supplementary material)**. The exclusion of outliers was not considered as there was no concern with the measurement of individuals, and it is believed that the exclusion of these cases may underestimate the true variability presented by the study sample.

**DISCUSSION**

We used parallel analysis to assess the dimensionality of the ATT-19 scale and exploratory factorial analysis with oblimin rotation to extract the factor loadings. We did not find studies evaluating the dimensionality of this scale for comparison with the literature. Factor 1 shows us social support and the perception of diabetes as something controllable, facing the shame of having diabetes, not saying you have the disease, thinking you have nothing to do and not having someone to talk to. Factor 2 is related to individuals' perceptions of how diabetes has changed their lives.

**CONCLUSION**

In conclusion, the ATT-19 questionnaire presented an adequate construct validity in our patient's sample, although adjustments were necessary. Unlike the original study^(10)^, our results suggested a structure of two factors and the exclusion of seven items from the scale. These aspects are important to ensure the quality of results when using the scale in practice, as well as for comparison with other future studies.

**REFERENCES**

1. Kaiser HF. An index of factorial simplicity. Psychometrika 1974;39(1):31-36. https://doi.org/10.1007/BF02291575.

2. Tabachnick BG, Fidell LS. Using multivariate statistics: International edition. Pearson 2012, 2013.

3. Cho SJ, Li F, Bandalos D. Accuracy of the parallel analysis procedure with polychoric correlations. Educ Psychol Meas 2009;69(5):748-759. https://doi.org/10.1177/0013164409332229.

4. Auerswald M, Moshagen Mn. How to determine the number of factors to retain in exploratory factor analysis: A comparison of extraction methods under realistic conditions. Psychol Methods 2019;24(4):468. https://doi.org/10.1037/met0000200.

5. Holgado–Tello FP, Chacón-Moscoso S, Barbero-García I et al. Polychoric versus Pearson correlations in exploratory and confirmatory factor analysis of ordinal variables. Quality & Quantity. 2010;44(1):153-166. https://doi.org/10.1007/s11135-008-9190-y.

6. Shapiro A, Ten Berge JMF. Statistical inference of minimum rank factor analysis. Psychometrika 2002;67(1):79-94. https://doi.org/10.1007/BF02294710.

7. Costello AB, Osborne J. Best practices in exploratory factor analysis: Four recommendations for getting the most from your analysis. Pract Assess Res Eval 2005;10(1):7. https://doi.org/10.7275/jyj1-4868.

8. Revelle W (2018) Psych: procedures for personality and psychological research. Northwestern University, Evanston.

9. Garrido LE, Abad FJ, Ponsoda V. A new look at Horn’s parallel analysis with ordinal variables. Psychol Methods 2013;18(4):454. https://doi.org/10.1037/a0030005.

10. Welch G, Beeney LJ, Dunn SM et al. The development of the diabetes integration scale: a psychometric study of the ATT39. Multivar Exp Clin Res 1996;11(2):75-88.

11. Hair JF, Black WC, Babin BJ et al. Multivariate data analysis, 8th edn., Cengage Learning EMEA, Andover. Hampshire, 2019.

| **Table S1. Supplementary material.** Factor loadings of the of ATT-19 items | | |
| --- | --- | --- |
| **Item** | **Factor 1** | **Factor 2** |
| Q1. If I didn't have diabetes, I would be a very different person |  | 0.71 |
| Q3. Having diabetes was the worst thing that happened in my life |  | 0.65 |
| Q4. Most people have difficulty adapting to having diabetes |  | 0.35 |
| Q6. It seems like there's not much I can do to control my diabetes | 0.61 |  |
| Q7. There is little hope of leading a normal life with diabetes | 0.69 |  |
| Q8. Proper diabetes control involves a lot of sacrifice and inconvenience |  | 0.46 |
| Q9. I try not to let people know that I have diabetes | 0.69 |  |
| Q10. Being diagnosed with diabetes is the same as being sentenced to a lifetime of illness |  | 0.81 |
| Q16. There's nothing you can do if you have diabetes | 0.71 |  |
| Q17. There is no one I can talk to openly about my diabetes | 0.79 |  |
| Q18. I believe I live well with my diabetes |  | 0.41 |
| Q19. I usually think it's unfair that I have diabetes and other people have very good health | 0.62 |  |
| Eigenvalue | 2.97 | 2.19 |
| Common Variance Explained | 58% | 42% |

# **
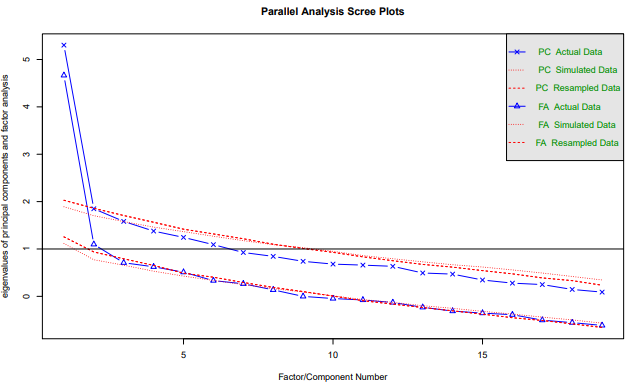
**

**Figure S1. Supplementary material. Parallel Analysis.**

PC = principal components; FA = factor analysis.

# **
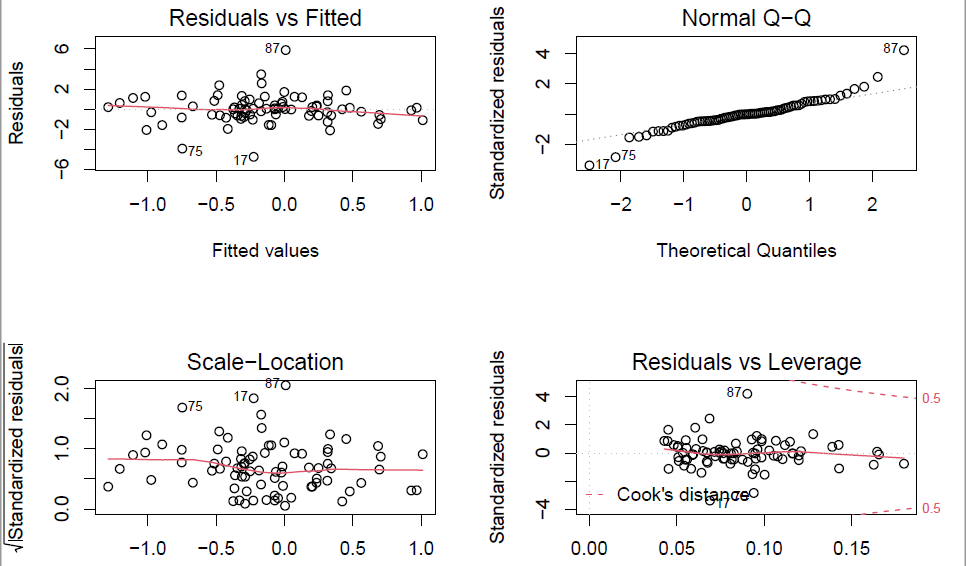
**

**Figure S2. Supplementary material. Regression Diagnostic**

| **Table S2. Supplementary material.** Proportion of patients who reported emotional and external eating attitudes (often/very often) according to positive/negative attitude towards diabetes (n=91) | | | |
| --- | --- | --- | --- |
|  | **Emotional Attitude toward Diabetes** | |  |
|  | **Positive attitude** | **Negative attitude** | **P^1^** |
| n | 23 | 68 |  |
| **Emotional Eating** |  |  |  |
| **Diffuse emotions** |  |  |  |
| Q3 | 2 (8.7%) | 16 (23.6%) | 0.12 |
| **Q8** | **2 (8.7%)** | **20 (29.4%)** | **0.04** |
| Q10 | 2 (8.7%) | 11 (16.2%) | 0.37 |
| Q28 | 2 (8.7%) | 12 (17.6%) | 0.30 |
| **Defined emotions** |  |  |  |
| **Q1** | **0** | **17 (25%)** | **0.008** |
| Q5 | 2 (8.7%) | 14 (20.6%) | 0.19 |
| Q13 | 2 (8.7%) | 7 (10.3%) | 0.82 |
| Q16 | 3 (13%) | 3 (4.4%) | 0.14 |
| Q20 | 4 (17.4%) | 18 (26.5%) | 0.37 |
| Q23 | 2 (8.7%) | 13 (19.1%) | 0.24 |
| Q25 | 2 (8.7%) | 13 (19.1%) | 0.24 |
| Q30 | 1 (4.3%) | 3 (4.4%) | 0.99 |
| Q32 | 2 (8.7%) | 10 (14.7%) | 0.46 |
| **External Eating** |  |  |  |
| Q2 | 6 (26.1%) | 21 (30.9) | 0.63 |
| Q6 | 5 (21.7%) | 17 (25%) | 0.75 |
| **Q9** | **2 (8.7%)** | **28 (41.2%)** | **0.004** |
| Q12 | 5 (21.7%) | 23 (33.8%) | 0.27 |
| Q15 | 2 (8.7%) | 19 (27.9%) | 0.05 |
| Q18 | 3 (13%) | 14 (20.6%) | 0.42 |
| **Q21** | **3 (13%)** | **30 (44.1%)** | **0.007** |
| Q24 | 3 (13%) | 22 (32.4%) | 0.07 |
| Q27 | 1 (4.3%) | 5 (7.4%) | 0.61 |
| Q33 | 2 (8.7%) | 11 (16.2%) | 0.37 |

Data are presented as the number of patients for total cases (%). ^1^ Chi-square test.
